# Role of Patch Testing With Standard and Cosmetic Series in Patients With Facial Allergic Contact Dermatitis Associated With Everyday Cosmetic Products

**DOI:** 10.7759/cureus.114953

**Published:** 2026-08-21

**Authors:** Kaveri Arora, Yuti Mishra, Paulina R Chaudhary

**Affiliations:** 1 Dermatology, Adesh Medical College & Hospital, Ambala, IND

**Keywords:** allergic contact dermatitis (acd), facial dermatitis, hair dye, indian cosmetic series, indian standard series, para-phenylenediamine, patch test

## Abstract

Background: Facial allergic contact dermatitis (ACD) is a common dermatological presentation, with rising incidence attributed to increasing cosmetic use. Patch testing remains the gold standard for identifying causative allergens. This study aimed to identify the allergens responsible for facial ACD using the Indian Standard Series and Indian Cosmetic Series.

Methods: A cross-sectional observational study was conducted on 30 patients aged 18-60 years with clinically diagnosed facial ACD attending the dermatology outpatient department of a tertiary care teaching hospital in Haryana, India. All patients underwent patch testing with 20 allergens of the Indian Standard Series and 32 allergens of the Indian Cosmetic Series, as per Contact and Occupational Dermatoses Forum of India (CODFI) recommendations. Readings were taken on Day 2 using International Contact Dermatitis Research Group (ICDRG) grading.

Results: The mean age was 39.26 ± 18.41 years, with female predominance (n=18,60%). The 41-50-year age group was most affected (n=11,36.67%). Homemakers constituted the largest occupational group (n=11,36.67%). Hair dye was the most commonly implicated agent (n=14,46.67%). The forehead (n=14,46.67%) and cheeks (n=12,40%) were the most affected sites. Itching and hyperpigmentation were the commonest clinical patterns (n=19,63.33% each). Para-phenylenediamine was the most frequent allergen (n=21), followed by fragrance mix (n=11), cetrimide (n=9), nickel sulphate (n=7), and parthenium (n=7).

Conclusion: Facial ACD has become common with the widespread use of cosmetic products. Patch testing plays a crucial role in confirming the diagnosis and identifying the specific allergens responsible for sensitization. Testing with both standard and cosmetic series increases the likelihood of detecting clinically relevant allergens that may be missed with standard series alone. Identification of the offending allergens enables individualized counseling regarding avoidance of culprit ingredients and selection of safer alternatives, thereby reducing recurrent episodes. Routine consideration of patch testing should therefore be encouraged in patients with persistent, recurrent, or treatment-resistant facial dermatitis with a suspected cosmetic etiology.

## Introduction

Contact dermatitis is an inflammatory skin reaction resulting from direct or indirect contact with noxious agents in the environment and is broadly divided into allergic and irritant types [1]. Allergic contact dermatitis (ACD) is a delayed type IV hypersensitivity reaction occurring in sensitized individuals and accounts for approximately 20% of all contact dermatitis, with a reported prevalence ranging from 1.7% to 6% [1,2]. Its incidence is rising owing to the increasing use of cosmetics and personal care products, with a corresponding burden of morbidity and impairment of quality of life [3,4].

The face is a common site of presentation, where exogenous and endogenous factors contribute to disease. Para-phenylenediamine, nickel, fragrance mix, and methylisothiazolinone are among the commonest sensitizing allergens [5]. Because cosmetic products are complex mixtures of chemical ingredients, clinical history alone is frequently insufficient to identify the offending agent [6].

Mechanistically, ACD develops in two phases. During sensitization, low-molecular-weight haptens bind cutaneous proteins and associate with major histocompatibility complex molecules on antigen-presenting cells, priming allergen-specific T cells in the regional lymph nodes [7]. On re-exposure, these primed T cells drive a rapid inflammatory response that produces the clinical phenotype. This delayed, T-cell-mediated mechanism distinguishes ACD from irritant contact dermatitis, in which inflammation is dose-dependent and immunologically non-specific, although the two frequently coexist and may be clinically indistinguishable [6].

Patch testing is the gold standard for diagnosing ACD; it reproduces a miniature model of the elicitation phase of delayed hypersensitivity by applying standardized allergens to the upper back, with readings taken at 48 and 96 hours as per International Contact Dermatitis Research Group (ICDRG) criteria [7,8]. In India, the Contact and Occupational Dermatoses Forum of India (CODFI) has standardized the Indian Standard Series and the Indian Cosmetic and Fragrance Series for routine use, reflecting the distinct exposure pattern of the Indian population [9]. Identification of the causative allergen is central to management, since avoidance of the culprit agent is the principal therapeutic goal. As facial skin is thin and highly exposed to cosmetics and airborne agents, the face is a particularly informative site for studying cosmetic-related sensitization. The present study was conducted to identify the allergens responsible for facial ACD by patch testing patients attending a tertiary care teaching center in North India.

## Materials and methods

This cross-sectional observational study enrolled 30 patients of either sex, aged 18-60 years, clinically diagnosed with facial ACD and attending the Department of Dermatology of a tertiary care teaching hospital (Adesh Medical College & Hospital, Ambala) in Haryana, India, over one year (1st June 2023 to 31st May 2024) following Institutional Ethics Committee approval.

The primary objective of the study is to identify the most common causative allergens in patients with facial ACD. The secondary objective is to determine the demographic profile of patients and to identify the various clinical patterns of patients with facial ACD.

Patients with clinically suspected facial ACD and a history of cosmetic use with features suggestive of an adverse cosmetic reaction were included. Patients receiving systemic corticosteroids (≥10 mg prednisolone equivalent) or other immunosuppressive therapy; those with sunburn; pregnant or lactating women; children; and non-consenting individuals were excluded. Written informed consent was obtained from all participants.

**Figure 1:**
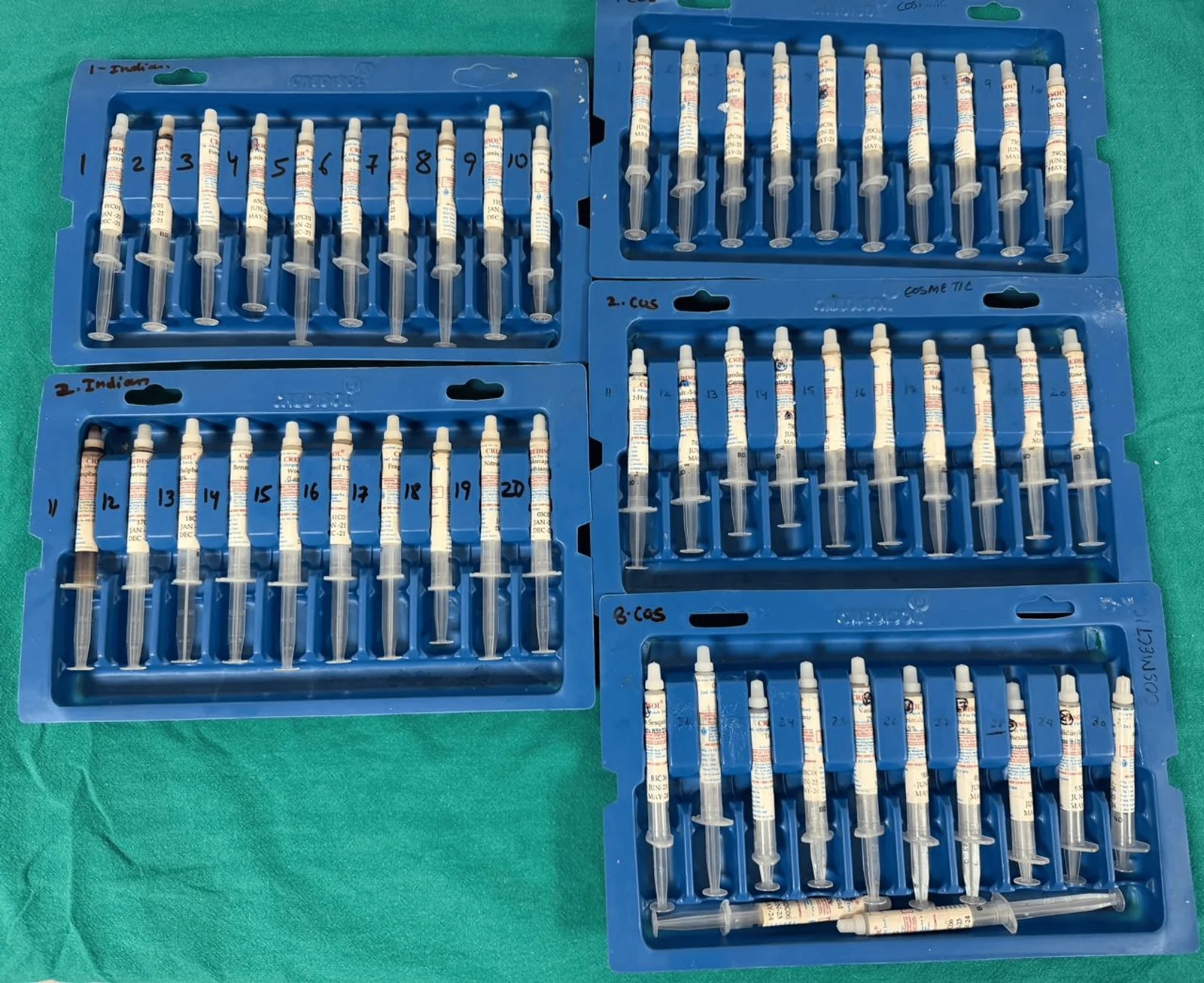
Patch Test Kits: Indian Standard & Cosmetic Series

**Figure 2:**
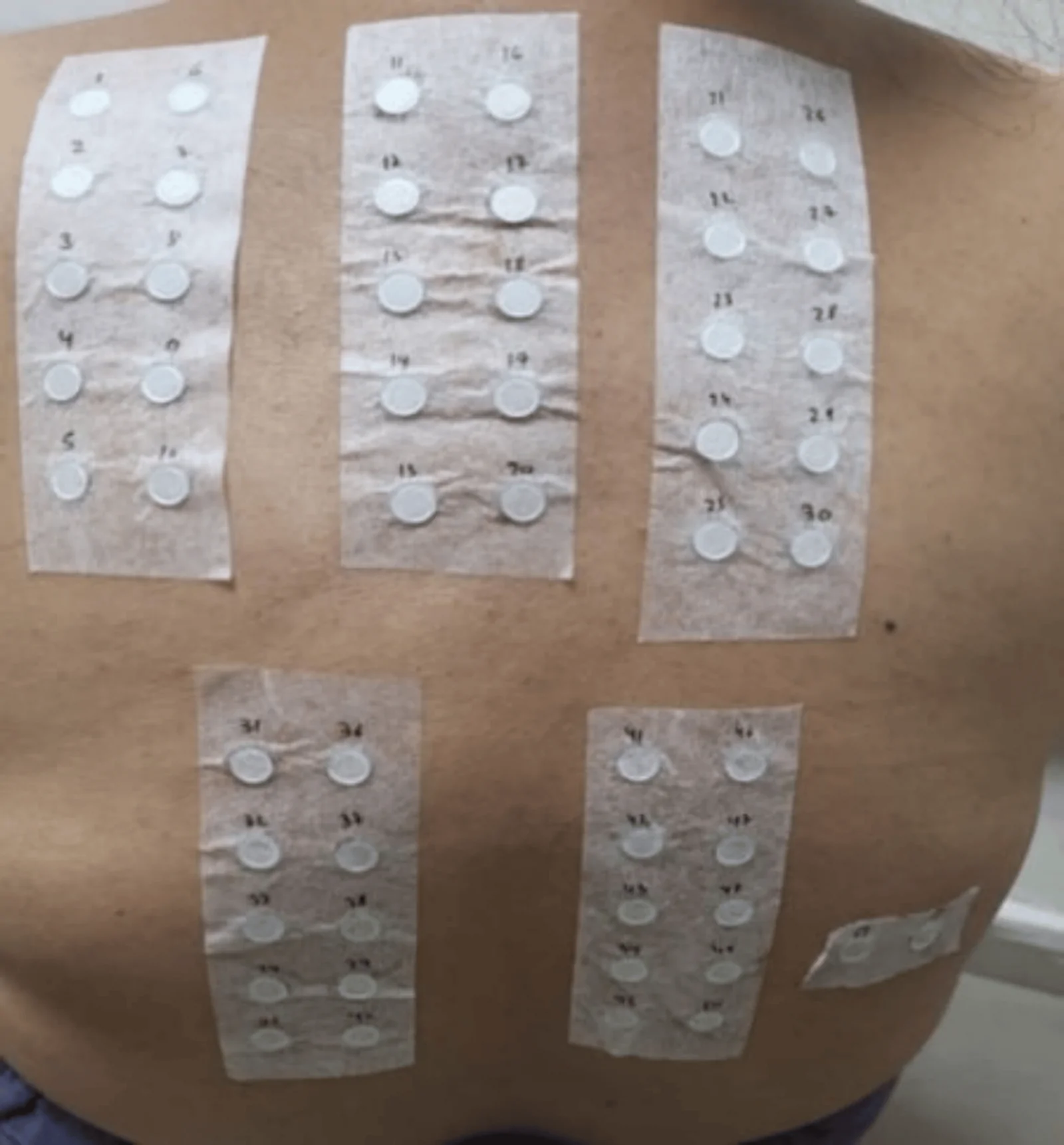
Patch Test Units Applied over Back

Demographic data and details of personal care product use, including type, frequency, and duration of application and any history of exacerbation, were recorded. Patch testing was performed using 20 allergens of the Indian Standard Series and 32 allergens of the Indian Cosmetic Series per CODFI recommendations, employing aluminum chambers (internal diameter 9 mm, depth 0.7 mm) mounted on microporous tape. Petrolatum-based allergens were placed directly in the chambers, while liquid allergens were applied on filter paper within the chambers. The units were fixed to the cleaned upper back, and the sites were registered on a patch test record form (Figures 1, 2).

The data collected were entered into a Microsoft Excel sheet, and statistical analysis was done using IBM SPSS Statistics for Windows, Version 28 (Released 2021; IBM Corp., Armonk, New York, United States). Categorical variables were presented as frequencies and percentages, and continuous variables were presented as mean and standard deviation. 

Patches were left in place for 48 hours. Patients were instructed to avoid tight clothing, scratching, wetting, sweating and ultraviolet exposure of the test area. Readings were taken 30 minutes after patch removal on Day 2 and graded according to the ICDRG system (Table 1) [8]. The patients were called on day 4 for any delayed reactions.

**Table 1:** ICDRG Grading of Patch Test Reactions ICGDRG: International Contact Dermatitis Research Group

Grade	Clinical finding	Interpretation
?+	Faint erythema only	Doubtful reaction
+	Erythema, infiltration, discrete papules	Weak positive (non-vesicular)
++	Erythema, infiltration, papules, vesicles	Strong positive (vesicular)
+++	Intense erythema, coalescing vesicles	Extreme positive (bullous)
–	No change	Negative reaction
IR	Irritant morphology like Necrosis, bullae	Irritant reaction

Patients with positive results were managed with topical corticosteroids, oral antihistamines, and saline soaks for acute weeping lesions, and counselled on allergen avoidance. 

## Results

Thirty patients with facial ACD were studied. The mean age was 39.26 ± 18.41 years, with the 41-50-year group most represented (n=11, 36.67%), followed by the 21-30-year group (n=8, 26.67%) and 31- 40-year age group (n=5,16.67%). Out of the study population, 18 patients (60%) were female and 12 patients (40%) were male. Educational status was highest among graduates and above (n=10, 33.33%) and higher-secondary level (n=9, 30%). Homemakers formed the largest occupational group (n=11, 36.67%), followed by those in salaried jobs (n=10, 33.33%) (Table 2)

**Table 2:** Demographic and Occupational Profile (n = 30)

Variable	Category	n	%
Age (years)	18–20	1	3.33
	21–30	8	26.67
	31–40	5	16.67
	41–50	11	36.67
	51–60	5	16.66
Sex	Female	18	60
	Male	12	40
Education	Illiterate	3	10
	Primary	3	10
	Secondary	5	16.67
	Higher secondary	9	30
	Graduate & above	10	33.33
Occupation	Homemaker	11	36.67
	Job	10	33.33
	Laborer	4	13.33
	Business	3	10
	Farmer	2	6.67

Disease onset was gradual in 24 patients (80%) and sudden in six patients (20%). The most common disease duration was 3-5 months (n=10,33.33%), followed by under one month (n=9,30%). A history of similar episodes in the past was the most frequent personal characteristic (n=14, 46.67%), followed by atopy (n=7, 23.33%) and seasonal variation (n=6, 20%).

The forehead was the most commonly affected site (n=14, 46.67%), followed by the cheeks (n=12, 40%) and nose (n=9, 30%). In some patients, multiple areas were involved; those using hair dye had involvement of the forehead along with the ears and nape of the neck. That's the reason why there were 51 responses in a sample size of 30.

Itching and hyperpigmentation were the predominant clinical patterns (n=19 each, 63.33%), followed by erythema (n=6,20%) (Table 3). Most of the patients presenting with facial ACD had multiple clinical patterns, such as itching along with erythema or itching along with hyperpigmentation. The sample size was 30 but collective responses were 52.

**Table 3:** Clinical Profile: Site, Implicated Product and Morphology (n = 30)

Variable	Category	n	%
Site	Forehead	14	46.67
	Cheeks	12	40
	Nose	9	30
	Lips	4	13.33
	Ears	3	10
	Face (diffuse)	3	10
	Chin	2	6.67
	Nape of neck	2	6.67
	Perioral	1	3.33
	Retro-auricular	1	3.33
Product/exposure	Hair dye	14	46.67
	Fairness cream	6	20
	Jewellery	5	16.67
	Lipstick	3	10
	Shaving cream	2	6.67
	Airborne allergen	2	6.67
	Cement/sindoor/mask/plant	1 each	3.33
Clinical pattern	Itching	19	63.33
	Hyperpigmentation	19	63.33
	Erythema	6	20
	Redness	5	16.67
	Lichenoid eruption	2	6.67
	Depigmentation	1	3.33

Hair dye was the most frequently implicated agent (n=14,46.67%), followed by fairness cream (n=6,20%) and jewelry (n=5,16.67%)

On Day 2 of Patch testing, Para-phenylenediamine produced the highest number of positive reactions (n=21; 8 graded +, 10 ++ and 3 +++), followed by Fragrance mix (n=11; 8 graded +, 3 ++), Cetrimide (n=9; 2 doubtful, 3 +, 3 ++,1 +++), Nickel Sulphate (n=7; 1 +, 5 ++, 1+++), Parthenium (n=7; 4 +, 3 ++) and Black rubber mix (n=6; 2 doubtful,4 +,). Gallate mix and Potassium dichromate each elicited reactions in 5 patients. Vaseline, Formaldehyde, Mercaptobenzothiazole, Epoxy Resin, Neomycin sulphate, Benzocaine, Thiuram mix and Nitrofurazone produced no reactions (Table 4). 

**Table 4:** Allergens Eliciting Positive Patch Test Reactions on Day 2, with Reaction Grades (n = 30)

Allergen	Total positive	?+	+	++	+++
Para-phenylenediamine	21	0	8	10	3
Fragrance mix	11	0	8	3	0
Cetrimide	9	2	3	3	1
Nickel sulphate	7	0	1	5	1
Parthenium	7	0	4	3	0
Black rubber mix	6	2	4	0	0
Gallate mix	5	1	1	2	1
Potassium dichromate	5	1	3	1	0
Musk mix	3	2	1	0	0
Rose oil	3	1	2	0	0
Propylene glycol	3	0	3	0	0
Colophony	2	0	0	1	1
Benzyl alcohol	2	0	2	0	0
Perubalsam	1	0	0	1	0
Cobalt sulphate	1	0	1	0	0
Wool alcohol	1	0	1	0	0
Chlorocresol	1	0	0	0	1
Chromium	1	0	1	0	0
Diazolidinyl urea	1	1	0	0	0
Ethylenediamine	1	0	1	0	0
Hexamine	1	0	1	0	0
Imidazolidinyl urea	1	0	1	0	0
Isopropyl myristate	1	0	0	1	0
Parabens mix	1	0	1	0	0
Thiomersal	1	0	0	0	1

In a sample size of 30, each patient reacted to patch testing in different ways. Out of 30 patients, four patients showed a positive reaction to one allergen, three patients reacted positively to two antigens, six patients reacted positively to three antigens and 17 patients reacted positively to four or more allergens. The severity of reaction was variable for different allergens ranging from doubtful to +++.

Reaction grading on day 2 was + for 47 allergens, ++ in 30 allergens, +++ in nine allergens and doubtful +? in 10 allergens, making a total of 96 positive patch test results.

Further follow-up could not be ensured as the patient faced accessibility challenges due to the rural location of the center. So the readings on day 4 could not be taken.

## Discussion

The present study aimed to examine the epidemiology, clinical presentation, and allergic triggers of ACD among a cohort of patients, shedding light on its demographic, occupational, and allergenic patterns. ACD remains a significant concern, with various factors such as age, gender, occupation, and environmental exposure influencing its prevalence and manifestation. The findings revealed that the condition is more common among individuals in the 41-50-year-age group, with a higher incidence in females as compared to males. Occupational factors also play a crucial role, with housewives and agriculturists being at higher risk of developing ACD due to constant exposure to allergens. The study identified several common allergens, including paraphenylenediamine, nickel, and potassium dichromate, with patch testing serving as an essential diagnostic tool. These allergens were found to be most prevalent in materials such as hair dyes, jewelry, and construction-related products. Additionally, the study highlighted the seasonal variation in ACD, with a higher incidence observed in hot and humid months. By examining these findings, we aim to deepen our understanding of the factors contributing to ACD, ultimately guiding more effective prevention and treatment strategies for affected individuals. 

In our study, the distribution of patients according to age showed the highest proportion in the 41-50 years age group, with 11 patients (36.67%) in that category. We observed that the maximum number of subjects in our study were female (n=18,60%) followed by male (n=12, 40%). In a study by Samanta et al., most patients were in the 4th decade, with a mean age of 39.7 years. This consistency highlights that middle-aged individuals are at increased risk, likely due to cumulative exposure to allergens over time [10]. Sharma et al. reported that most cases were seen in the 30-44 years age group (44.1%), followed by 45-59 years (34.9%), and documented 77 female and 29 male patients [11]. Tramontana et al. also highlighted that ACD is commonly seen in adults, especially in the middle-aged population due to prolonged allergen exposure [3]. Madhumitha et al. reported the mean age was 40.4 ± 13.7 years with the male-to-female ratio of 3:2. It had slight male predominance in their study, though other studies noted either gender parity or female predominance [12]. Rastogi et al. reported a mean age of 47 years, with patients ranging from 16 to 78 years and a female preponderance of 64%, with housewives forming the majority [13].

The distribution of patients according to education revealed that the largest group consisted of those with graduate and higher education, comprising (n=10) 33.33% of the patients. This was followed by individuals with higher secondary education, secondary education, primary education, and illiterate groups. This trend might reflect increased awareness and reporting of dermatological conditions in this subgroup. The educational background could also influence exposure patterns, with professionals more likely to use a variety of cosmetic and occupational products that predispose them to ACD. This parameter has not been widely studied, making our findings a valuable addition to understanding the socio-educational dynamics of ACD.

On the basis of the onset of disease, the distribution of patients revealed that the majority experienced a gradual onset, with 24 patients (n=24, 80%). In contrast, six patients (n=6, 20%) had a sudden onset of disease. The distribution of patients according to the duration of disease showed that the highest proportion, 10 patients (n=10,33.33%), had a disease duration of 3-5 months. Samanta et al. found that 89.5% of patients reported an insidious onset and noted a higher duration of 1-2 years (53%). Our study results contrast with the results of Samanta et al. These differences may stem from variations in healthcare-seeking behavior and environmental exposure [10].

The most common site involved was the forehead (n=14, 46.67%), followed by the cheeks (n=12,40%), nose (n=9, 30%), lips (n=4,13%), ears (3,10%), and nape of the neck (2,6.67%). Perioral and retroauricular regions were the least involved. Samanta et al. reported the forehead (76%) and medial cheeks (71%) as frequently involved sites, which were similar to the most common site in our study. These regions' exposure to cosmetics and environmental allergens underscores their vulnerability [10]. Rastogi et al. reported the forehead (50%), periorbital region (38%), and cheeks (30%) as the main sites. Similarly, Sharma et al. found facial involvement in a significant number of cases. These findings underscore the high exposure of facial skin to cosmetic allergens [11,13].

**Figure 3:**
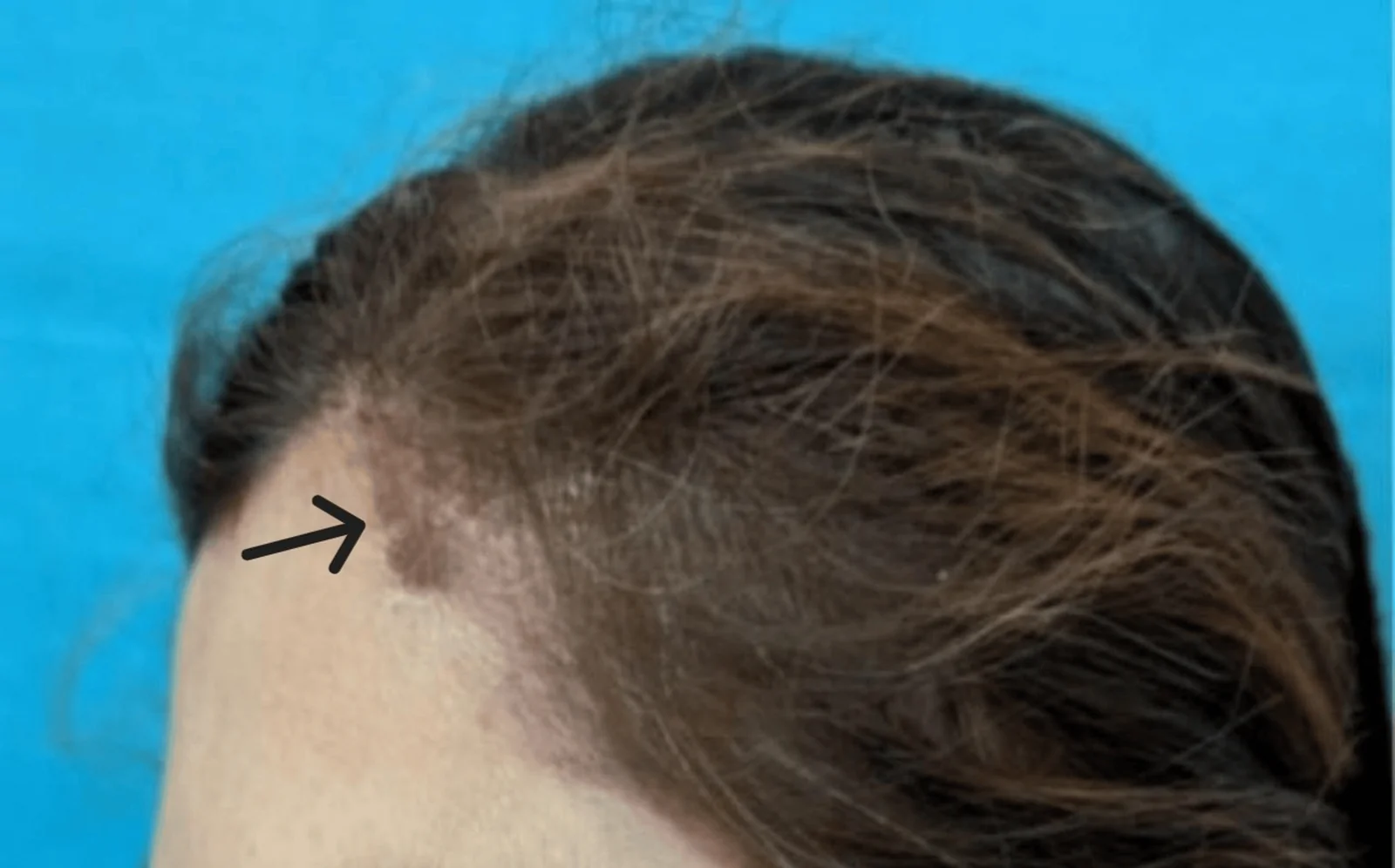
Allergic Contact Dermatitis to Hair Color

**Figure 4:**
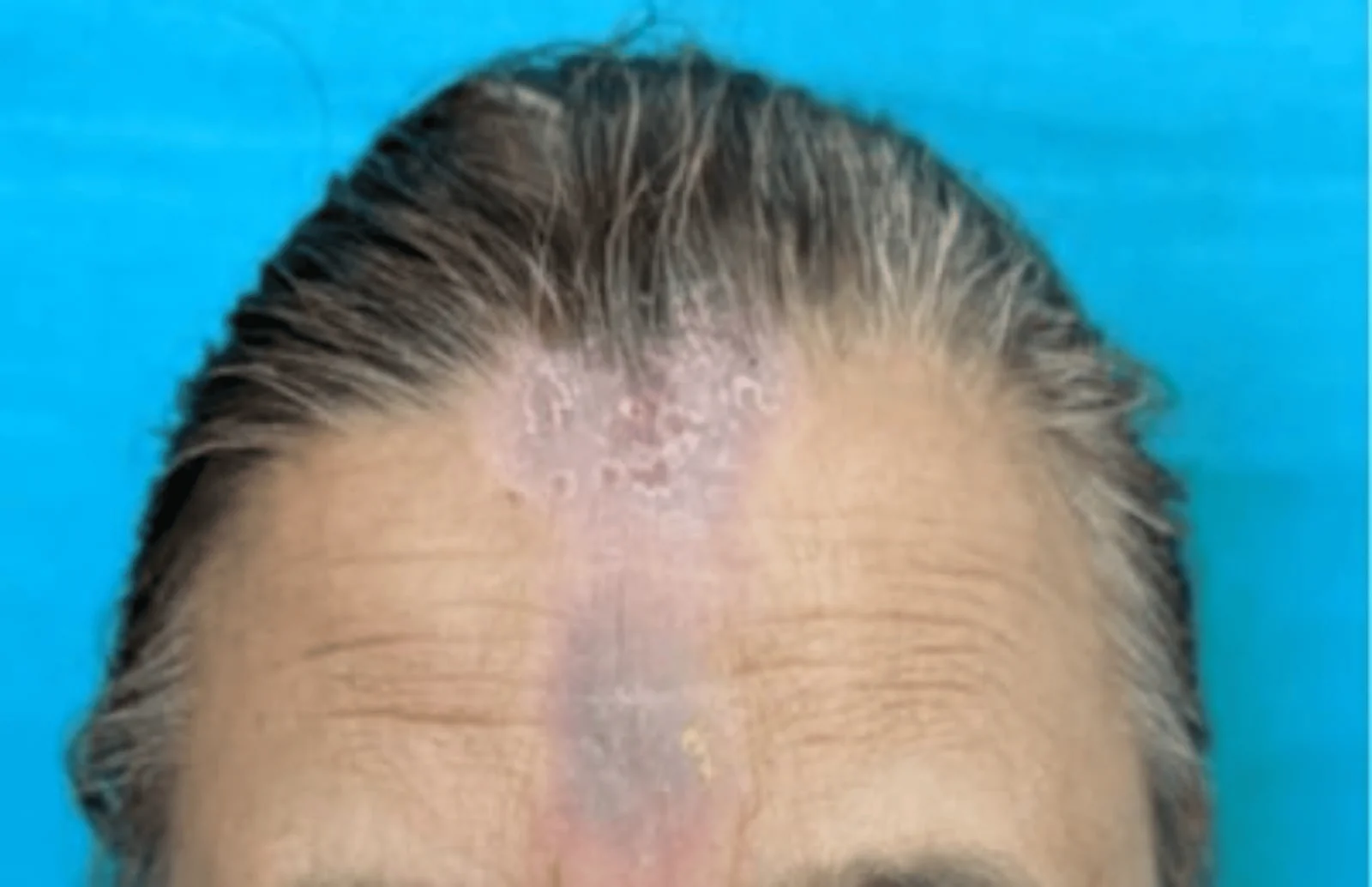
Allergic Contact Dermatitis Due to Application of Sindoor

According to the product used, the distribution of patients revealed that hair dye was the most common agent (Figure 3). This was followed by fairness cream, jewelry, shaving cream, and airborne allergens. Other exposures included lipstick, cement, sindoor, masks, and plants (Figure 4). Samanta et al. found that 74% of patients showed relevance to hair dye, particularly para-phenylenediamine (PPD). Fairness creams also emerged as significant allergens in both studies, underscoring the role of cosmetic agents in ACD [10]. In a study by Tramontana et al., para-phenylenediamine (PPD), a common hair dye component, was identified as a significant allergen [3]. Rastogi et al. also identified paraphenylenediamine (PPD) as the most common allergen (18%), followed by nickel sulfate (16%), thimerosal (16%), and fragrance mix (10%). Notably, Rastogi et al. found the Indian Standard Series to yield a higher positivity rate than the cosmetic series, underlining the value of testing both panels concurrently rather than either alone [13]. Sharma et al. also reported PPD as the most frequent allergen, along with cetrimonium bromide and thimerosal [11]. These findings align with the findings in our study. Sahu et al. found potassium dichromate, parthenium, and PPD leading to a 69% patch-test positivity rate in Eastern India, while Ünal et al. identified nickel sulphate and fragrance mix as predominant sensitizers in a large Turkish cohort, and Verma et al. reported thiomersal and cetrimide as common sensitizers in patients with melasma [14-16]. A retrospective study (2018) by Shariff et al. was conducted in South India to estimate the incidence of various allergens among 150 patch test-positive patients with ACD, and it was found that ACD to cement was the commonest (44.7%), followed by nickel (10%) and plant antigen (9.3%) [17]. Madhumitha et al. similarly observed nickel producing predominantly weak reactions and potassium dichromate stronger reactions, mirroring the graded responses seen here [12]. Kumar et al. observed hair dyes, soaps, shaving creams, and shampoos as the most frequent allergens, with 80% of hair dye users reporting ACD to hair dye. Additionally, itching was noted in roughly 90% of both sexes and photosensitivity in 42% of cases, predominantly among men, reflecting sex-linked differences in cosmetic exposure [18].

Collectively, these findings confirm that a regionally appropriate combination of the Indian Standard and Cosmetic Series captures the majority of relevant facial sensitisers and supports the routine use of comprehensive allergen panels. From a management perspective, the dominance of PPD-containing hair dye and fairness creams among implicated products identifies clear, modifiable targets for patient counseling. Because cosmetic formulations evolve with changing social and cultural trends, allergen batteries require periodic updating; a proportion of patients with a convincing exposure history may still test negative on existing panels, indicating sensitisation to agents not yet represented in standard series [19]. In an Indian study by Samal et al., in 97 patients, face cream was the most common allergic cosmetic (24.7%) causing ACD. The most common patch chemicals identified in the study were para-phenylinediamine (17.5%) followed by thiomersal (13.4%) [20]. In a study in South India by Mohan et al, the most common allergen with a positive patch test was fragrance mix (n=19, 25.7%) followed by PPD (n=18, 24.3%) and thiomersal (n=10, 13.5%). It is generally accepted that the leading cause of ACD associated with cosmetics is fragrance, followed by preservatives and PPD in hair dyes [21].

Patch test results

**Figure 5:**
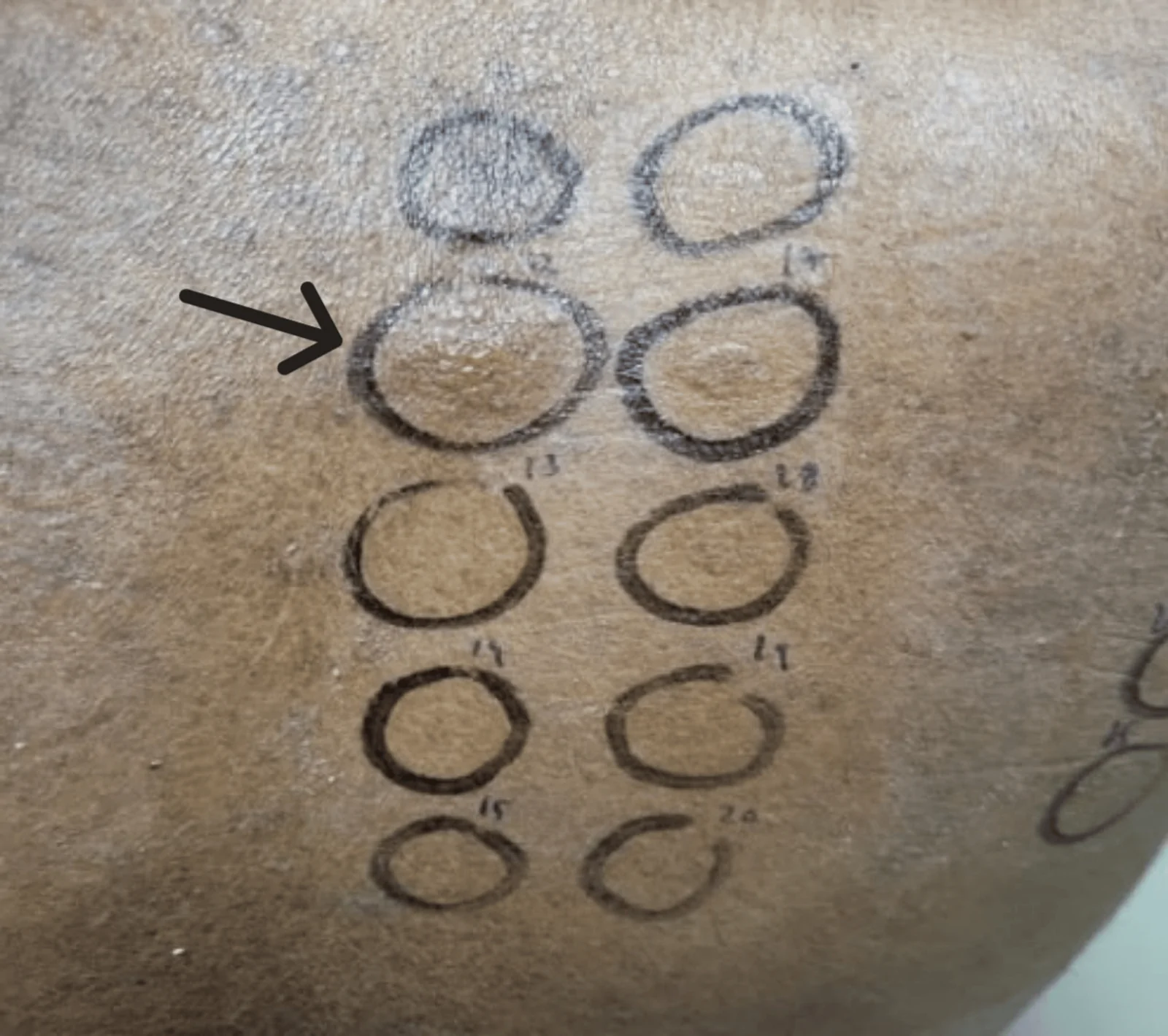
Patch Test Result on Day 2 Positive for PPD PPD: para-phenylenediamine, as shown by the arrow.

**Figure 6:**
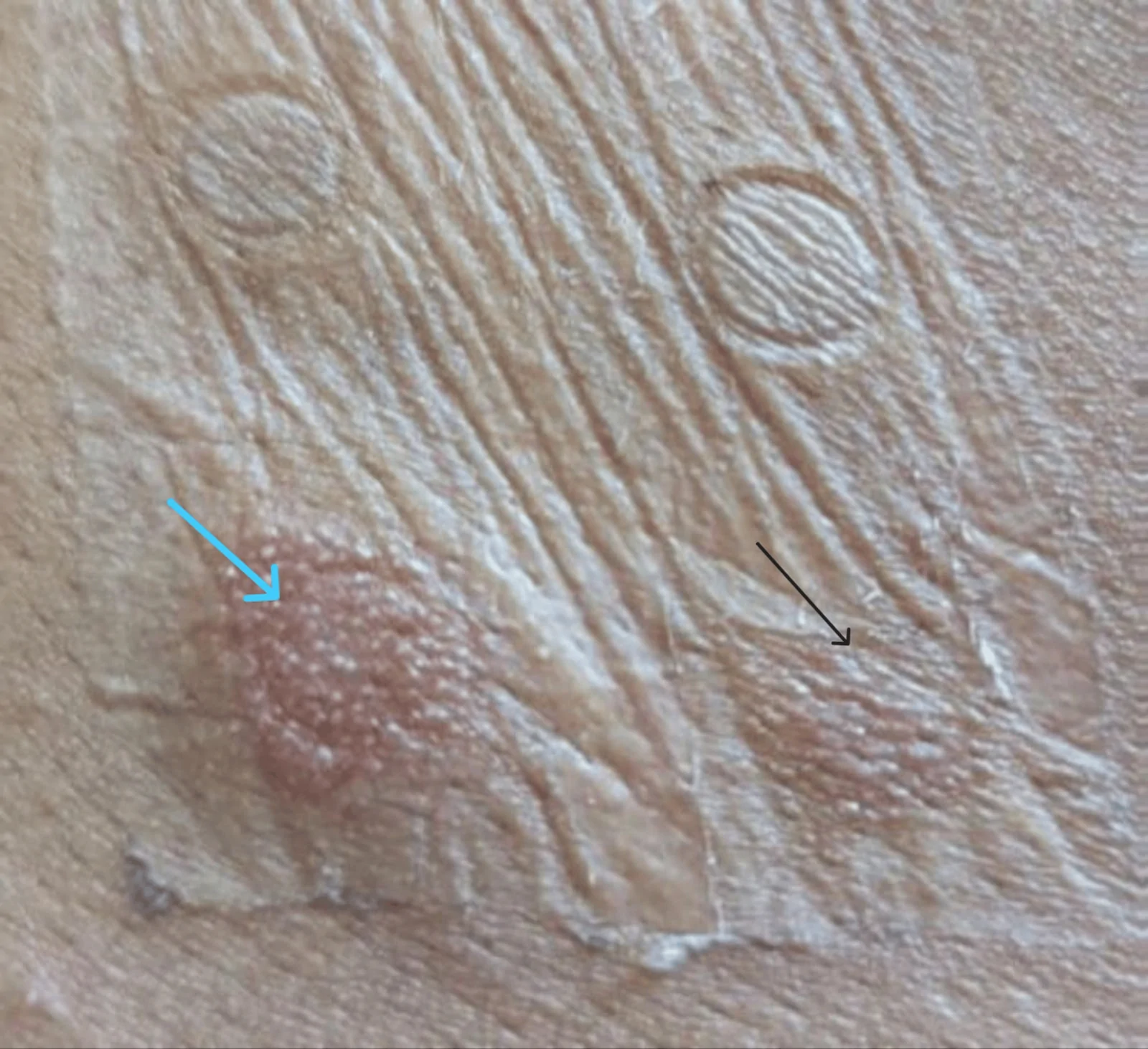
Patch Test Result on Day 2 Positive for Nickel (++) Represented by a Blue Arrow and Parthenium (+) Represented by a Black Arrow

In our study, the distribution of patients according to the grading of dermatitis on Day 2 after patch testing revealed significant variations in response to allergens. Para-phenylenediamine showed the highest number of positive reactions (Figure 5), with 21 patients exhibiting varying grades: eight patients with "+", 10 with "++", and 3 with "+++". In our study population, 14 patients had a history of hair dye application with positive patch test results, but 21 patients showed positivity to PPD in Patch Testing as few patients showed positivity to more than one allergen. Fragrance mix also showed notable positivity, with 11 patients, including eight patients with "+", and 3 with "++". Nickel sulphate elicited reactions in seven patients, predominantly as "++" (five patients) and "+++" (one patient) (Figure 6). Similarly, Parthenium caused positive reactions in seven patients, primarily with "+" in four patients and "++" in three patients. Black rubber mix and Cetrimide each caused reactions in six and nine patients, respectively, with varied intensities, while Gallate mix and potassium dichromate caused reactions in five patients each, mostly as "+". Other allergens such as rose oil, propylene glycol, and benzyl alcohol showed positive reactions in smaller numbers, mostly at lower intensities. Vaseline and many other allergens like formaldehyde and epoxy resin did not elicit any reactions.

The findings of the various studies align with the findings of our study, where common allergens yielded positive patch tests with varying strengths, and the most common allergen was para-phenylenediamine, which is present in hair dyes, followed by fragrance mix, nickel sulphate, and cetrimide.

Limitations

Our study has certain limitations that must be acknowledged. Being a single-centered study, the findings may not be generalizable to broader populations with varied environmental and socio-cultural exposures. Further follow-up could not be ensured as the patient faced accessibility challenges due to the rural location of the center. So the readings on day 4 could not be taken.

## Conclusions

Our study highlights the diverse clinical and demographic profile of patients presenting with facial ACD and underscores the importance of a targeted diagnostic approach. Facial ACD has become increasingly common with the widespread use of everyday cosmetic products. Patch testing, particularly with both Indian Standard and Cosmetic series, is an important diagnostic tool for identifying clinically relevant allergens that may otherwise remain unrecognized. Identification of the causative allergens facilitates individualized counseling regarding allergen avoidance and selection of safer cosmetic alternatives, thereby reducing recurrence and improving long-term outcomes. These findings reinforce the value of using regionally relevant allergen series for accurate allergen identification and targeted management of facial ACD. Increased awareness regarding the potential risks associated with commonly used cosmetics, as well as appropriate allergen avoidance, is essential. Considering the contribution of environmental, occupational, and personal exposures, patient education should form an integral part of management. Larger, multicentric studies involving diverse populations are warranted to further characterize relevant sensitization patterns and strengthen evidence-based strategies for prevention and management of cosmetic-related facial ACD.
